# Cooperative Intersection with Misperception in Partially Connected and Automated Traffic

**DOI:** 10.3390/s21155003

**Published:** 2021-07-23

**Authors:** Chenghao Li, Zhiqun Hu, Zhaoming Lu, Xiangming Wen

**Affiliations:** 1 Beijing Laboratory of Advanced Information Networks, Beijing University of Posts and Telecommunications, Beijing 100876, China; lichenghao@bupt.edu.cn (C.L.); lzy_0372@163.com (Z.L.); xiangmw@bupt.edu.cn (X.W.); 2 School of Computing and Information Engineering, Hubei University, Wuhan 430062, China

**Keywords:** connected and automated vehicles, signalized intersection, perceptual error

## Abstract

The emerging connected and automated vehicle (CAV) has the potential to improve traffic efficiency and safety. With the cooperation between vehicles and intersection, CAVs can adjust speed and form platoons to pass the intersection faster. However, perceptual errors may occur due to external conditions of vehicle sensors. Meanwhile, CAVs and conventional vehicles will coexist in the near future and imprecise perception needs to be tolerated in exchange for mobility. In this paper, we present a simulation model to capture the effect of vehicle perceptual error and time headway to the traffic performance at cooperative intersection, where the intelligent driver model (IDM) is extended by the Ornstein–Uhlenbeck process to describe the perceptual error dynamically. Then, we introduce the longitudinal control model to determine vehicle dynamics and role switching to form platoons and reduce frequent deceleration. Furthermore, to realize accurate perception and improve safety, we propose a data fusion scheme in which the Differential Global Positioning system (DGPS) data interpolates sensor data by the Kalman filter. Finally, a comprehensive study is presented on how the perceptual error and time headway affect crash, energy consumption as well as congestion at cooperative intersections in partially connected and automated traffic. The simulation results show the trade-off between the traffic efficiency and safety for which the number of accidents is reduced with larger vehicle intervals, but excessive time headway may result in low traffic efficiency and energy conversion. In addition, compared with an on-board sensor independently perception scheme, our proposed data fusion scheme improves the overall traffic flow, congestion time, and passenger comfort as well as energy efficiency under various CAV penetration rates.

## 1. Introduction

The increasing vehicle volume has profoundly affected our social life in terms of mobility, safety, and environmental pollution. Studies show that about 99 h a year are wasted on the road due to congestion, costing them nearly $88 billion in 2019, an average of $1377 per person per year in the USA [1]. Meanwhile, an estimated 38,800 people lost their lives in 2019 due to car crashes [2], where more than 90% of accidents are caused by human errors [3]. On the other hand, the emission of automobile exhaust is the biggest direct source of USA greenhouse gas [4]. Fortunately, the emerging of connected and automated vehicles are recognized as one of the promising solutions to improve road traffic safety and efficiency [5].

Currently, with the rapid development of on-board sensors devices (e.g., LiDAR, Radar, and camera) and wireless communication technologies (e.g., Dedicated short range communication and 5G), the vehicle has the potential to sense the surrounding environment and cooperate with other vehicles to extend the sensing range [6]. Specifically, through reliable vehicle-to-vehicle (V2V) and vehicle-to-infrastructure (V2I), information can be shared among vehicles and infrastructures, which allows vehicles to coordinate their maneuvers for platooning and achieve efficiency goals.

Related works have already been conducted on getting through the intersection quickly and safely for CAVs. In [7], a cooperative autonomous traffic organization method for CAVs in multi-intersection road networks is proposed, where a multi-objective trajectory optimization is used to resolve conflicts and ensure efficient and comfortable trips. The author in [8] proposed a virtual platoon based distributed control framework to enable the vehicles to stagger through the intersection, where the conflict-free topology was presented according to the relationship of traffic order of vehicles. In [9], a decentralized optimal control is presented to investigate the associated trade-off between minimizing fuel consumption and passenger discomfort while the vehicle turns at the intersection. Tallapragada et al. introduced a hierarchical distributed coordination structure in [10] which use a central intersection manager to communicate with vehicles heading towards the intersection and group them into clusters to optimize their individual trajectories. Zhang et al. proposed a decentralized framework to implement trajectory planning for the vehicles at a signal-free intersection, where the trade-off between energy and travel time is quantified and safety constraints are incorporated [11]. In [12], a distributed linear controller is designed to organize vehicles’ movements with the proposed conflict-free geometric topology, where the vehicles are projected into virtual platoons. In these studies, CAVs control their trajectory according to the movement information of neighboring vehicles, where the frequent interaction of information between vehicles and the ability of online computation are required. Vehicle platoon is mentioned in many works to improve traffic efficiency and the traffic performance can be further improved by vehicle–infrastructure cooperation.

Indeed, CAVs can receive the Signal Phase and Timing (SPaT) information from the signalized intersection in advance to control their maneuvers, which can avoid congestion and reduce the number of stops. Green Light Optimized Speed Advisory or eco-driving was proposed to implement a collaboration between vehicles and intersections [13,14,15,16]. Nunzio et al. proposed the eco-driving algorithm which assists the vehicle in passing through multiple intersections in succession with green lights [17]. Yang et al. proposed a cooperative adaptive cruise control system to ensure that the vehicle arrives at the stop bar as the last vehicle has just passed through the intersection, which can reduce congestion and emissions [18]. The authors in [19] developed a platoon-based trajectory optimization method which controls the leading vehicle in each platoon to minimize the fuel consumption and ease traffic congestion. In [20], a cooperative eco-driving system for signalized intersections was presented and the effect of CAV penetration rate on the traffic efficiency was analyzed. In [21], a distributed and cooperative eco-driving method was proposed to improve the traffic efficiency under mixed traffic flow.

However, the cooperation between vehicles requires accurate sensing data, and the error free perception is assumed in the above works. Errors may occur in the sensing process of vehicle on-board sensors due to the weather, obstacles, the driving style of the operator, or vehicle control algorithm [22]. In addition, CAVs and conventional vehicles will coexist in the near future and imprecise perception needs to be tolerated in exchange for mobility. In [23], random Gaussian white noise is added to the intelligent driver model, and the results show that the speed flow instability from the noise causes far more fluctuations in the fleet than the introduced noise. The author in [24] also introduced perceptual errors to the vehicle following model and found that the errors were more likely to introduce instability in the ride during vehicle acceleration. In [25], the authors propose that adding random offsets to the ideal velocity values calculated by the model can help the control system better cope with the real environment. Furthermore, the interaction between perceptual error and time headway on vehicles driving freely was studied in [26], and the results show that a smaller headway time distance exacerbates the effect of perceptual errors. The effect of vehicle perception error under multiple intersections was studied in [27], and the model captures the real world trade-off between safety and efficiency for potential future traffic systems. On the other hand, related works have already been studied on improving the accurate perception of the vehicle. The authors in [28] proposed a single road side unit (RSU) based vehicle localization algorithm which uses linear least squares type methods in a close-form manner. The RSU-based localization information was integrated with the vehicle kinematics information to improve the accuracy of vehicle localization in [29]. In [30], a vehicle positioning scheme was developed on the basis of a beacon message broadcast periodically by multi-RSUs. Notably, due to the limitation of the RSU communication range, multiple RSUs need to be deployed to locate the vehicle continuously, which require additional resources, especially when the road is long. On the other hand, the vehicle can receive its real-time position via a Differential Global Positioning system without additional road equipment, which is more accurate but slower to update than vehicle sensors [31]. The vehicle positioning algorithm was introduced in [32,33] based on low-cost GPS and sensors. In [34,35,36], the DGPS data were fused with vehicle perception data by the Kalman filter to obtain the precise vehicle motion state estimation. However, the update frequency of DGPS data is slow, and the perception data may be outdated, which cannot meet traffic demands at urban intersections. Finally, due to the low penetration of CAV on the roads, there is no actual empirical data to show the performance of cooperative signalized intersection in error environments.

The main contributions of this paper are as follows:

- We introduce a simulation model to study the effect of vehicle perceptual error and time headway at cooperative signalized intersection, where the OU random process is used to extend the IDM model and describe perceptual error, which can lead to accidents due to inaccuracy perception data and make the car follow model more realistic.
- We present the vehicle longitudinal control model to describe their dynamics and determine their roles based on their distance to intersection, where vehicles can switch between leader and follower to reduce frequent deceleration.
- We propose a data fusion scheme in which the DGPS data interpolate on-board sensor data by the Kalman filter to mitigate the effect of perceptual error and achieve timely data updates.

According to the simulation, the trade-off between the traffic efficiency and safety at intersection is found that lower time headway and serious perceptual error will lead to congestion and traffic accidents due to the inaccurate speed control and strong speed fluctuation. On the other hand, excessive time headway will result in low traffic efficiency and energy conversion. The results also show that our proposed data fusion scheme significantly improves the traffic performance in terms of safety, efficiency, as well as passenger comfort under various CAV penetration rates.

The remainder of this paper is organized as follows: Section 2 illustrates the overall scenario and longitudinal control model. Section 3 describes the vehicle perception data processing system. Section 4 introduces the evaluation indicators of traffic performance. A simulation of the proposed scheme has been conducted, and its result are evaluated and analyzed in Section 5. Section 6 concludes this paper.

## 2. System Model

**Figure 1:**
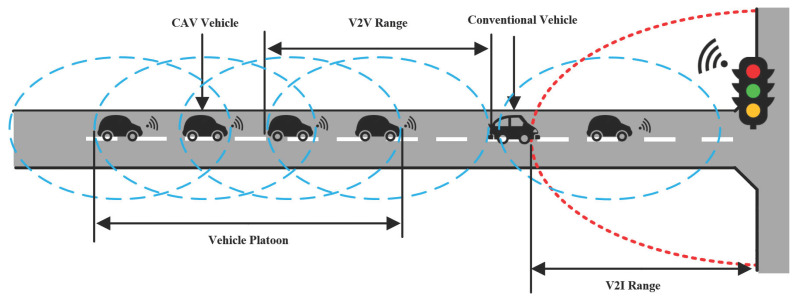
Illustration of the traffic intersection system.

As shown in Figure 1, we consider a typical intersection with a one-lane road where the lane length is *L* meters. For simplicity, we introduce a time variable $t_{i,0}$ to indicate the initial time that the *i*-th vehicle enters the road. Let $x_{i}\left(t\right)$ represent the position of the *i*-th vehicle’s midpoint at time *t*. To reduce the number of stops in the intersection, the vehicles are expected to pass through the intersection within its available green window. The available green window is determined by the current signal phase and the time when the vehicle receives the SPaT information. For example, if the current signal phase is green, the available green window is defined as $T_{g\_available}=\left[t_{i},t_{curr\_e}\right)\cup \left[t_{next\_s},t_{next\_e}\right)$, where $t_{i}$ and $t_{curr\_e}$ denote the current time when the vehicle *i* receives information from RSU and the end time of the current green window, respectively. $t_{next\_s}$ and $t_{next\_e}$ denote the start time and the end time of the next green window, respectively. On the other hand, if the current signal phase is red, the available green window is $T_{g\_available}=\left[t_{next\_s},t_{next\_e}\right)$. Note that the signals are fixed-timing controlled, where the switching time of green light and red light is set as $T_{switch}$.

The traffic at the intersection may contain conventional vehicles (e.g., manual vehicle) and automated vehicles (e.g., CAV). In particular, the conventional vehicle is assumed to be driven by human drivers without any ability to access to information in advance, and the driver’s visual range is limited to *H* meters. However, due to the on-board sensors (e.g., radar, controller) and dedicated short range wireless communication devices, CAVs have the ability to calculate the distance from neighbor vehicles and obtain its own driving data including speed and acceleration [6]. Additionally, CAVs can obtain the current and next period’s signal timing scheme from the road side infrastructure by V2I communication. The information can be shared with each other by V2V communication to develop platoons. The automated vehicle platoon consists of CAV leader and followers, which adopt different longitudinal control model for motion. In this part, we mainly focus on the longitudinal control model for leaders and followers, respectively.

### 2.1. Leaders’ Longitudinal Control Model

When the leader vehicle obtains the SPaT information from the intersection, the vehicle could adjust its speed to pass through intersections within the available green window accordingly. Based on the green phase information, there are three scenarios including the cruise scenario and the acceleration or deceleration scenario, as well as the stop scenario.

#### 2.1.1. Cruise Scenario

Once vehicles can travel through the intersection during the green phase without any speed change, then the scenario is a cruise scenario. Therefore, the vehicle maintains its velocity, and the longitudinal acceleration is zero.

#### 2.1.2. Acceleration or Deceleration Scenario

When the green light is short, the vehicle needs to accelerate and decelerate to pass through the intersection within the current or the next green light, respectively. Based on the SPaT information, we can derive the time that the vehicle arrives the intersection, denoted by $t_{arr}$. Then, we need to choose an acceleration or deceleration profile for which the vehicles are expected to reach the intersection at a specific time $t_{arr}$. To this end, a piecewise trigonometric-linear [37] algorithm is used to control the longitudinal movements of the vehicle, which can bring a smoother change in acceleration with low jerk and reduce the fuel consumption. The vehicle’s trigonometric acceleration/deceleration process is divided into two periods. Let $t_{i,s}$ be the time that vehicle *i* starts the acceleration/deceleration process. Then, the acceleration/deceleration can be defined by

(1) $$a_{tri}\left(t\right)=\left\{\begin{array}{c} v_{d}\cdot j\cdot \sin\left[j\cdot \left(t-t_{i,s}\right)\right],t\in \left[t_{i,s},\frac{\pi }{2j}+t_{i,s}\right)\\ v_{d}\cdot j\cdot \sin\left[k\cdot \left(t+\frac{\pi }{k}-\left(\frac{\pi }{2j}+\frac{\pi }{2k}+t_{i,s}\right)\right)\right],\\ t\in [\frac{\pi }{2j}+t_{i,s},\frac{\pi }{2j}+\frac{\pi }{2k}+t_{i,s}) \end{array}\right.$$

where $v_{h}=\frac{d}{t_{arr}-t_{i,s}}$, denoting the desired average speed to reach the intersection at given target arrival time $t_{arr}$, and *d* is the current distance of vehicle to the intersection; $v_{d}$ represents the difference between current speed and desired average speed, given by $v_{d}=v_{h}-v_{i}\left(t_{i,s}\right)$, where $v_{i}\left(t_{i,s}\right)$ is the instantaneous speed at current time instant $t_{i,s}$.

For Equation (1), *k* and *j* are the parameters to control the changing rate of acceleration or deceleration. Since the vehicle dynamics should be constrained by the vehicle powertrain’s ability and the comfort requirements of passengers, then *k* is chosen as the maximum that satisfies:

(2) $$\left\{\begin{array}{c} |k\cdot v_{d}|⩽a_{\max}\\ |k\cdot v_{d}|⩽-a_{\min}\\ |k^{2}\cdot v_{d}|⩽jerk_{\max}\\ k⩾\left(\frac{\pi }{2}-1\right)\cdot \frac{v_{h}}{d} \end{array}\right.$$

and

(3) $$\begin{array}{c} j=\frac{-\frac{\pi }{2}k-\sqrt{{\left(\frac{\pi }{2}k\right)}^{2}-4k^{2}\cdot \left[\left(\frac{\pi }{2}-1\right)-\frac{d}{v_{h}}\cdot k\right]}}{2[\left(\frac{\pi }{2}-1\right)-\frac{d}{v_{h}}\cdot k]} \end{array}$$

where $a_{max}$ and $a_{min}$ are maximal acceleration and deceleration, respectively. $jerk_{max}$ is the maximum jerk a passenger can stand.

#### 2.1.3. Stop Scenario

When the vehicle cannot avoid the red phase by either accelerating or decelerating, the vehicle should drive to the intersection by moderate deceleration to full stop while approaching the intersection. The reference deceleration is calculated by Equations (1)–(3), with

(4) $$\begin{array}{c} v_{h}=\frac{v_{i}\left(t_{i,s}\right)}{2} \end{array}$$

(5) $$\begin{array}{c} k=j=\frac{v_{h}}{d}\cdot \pi \end{array}$$

### 2.2. Followers’ Longitudinal Control Model

In a cooperative manner, a group of vehicles form a platoon where a leader guides the platoon on the road and the followers follow their preceding vehicles with a minimum safety distance to avoid crashes. To this end, a mathematical model called an intelligent driver model [38] was proposed to capture the dynamics of the follower vehicle in the platoon. However, the IDM model assumes that the maximal deceleration is unbounded. Consequently, the vehicle dynamics described by the model may execute unrealistic emergency braking maneuvers and stop immediately when they encounter dangerous situations, which is impossible in the real world due to the limitation of vehicle engines.

In addition, the IDM model considers vehicle motion in an error free environment. In practice, errors may occur when the information about positions and velocities is obtained. The reason is that the sensor is significantly influenced by the weather or driving environment. To model vehicle behavior under these random perceptual errors, the IDM extension model was presented by bounding the maximal deceleration and introducing the random misperception, which can be given by [26]

(6) $$\left\{\begin{array}{c} \frac{d}{dt}x_{i}\left(t\right)=max\left\{v_{i}\left(t\right),0\right\}\\ \frac{d}{dt}v_{i}\left(t\right)=max\left\{a_{\max}\cdot \left(1-{\left(\frac{v_{i,error}\left(t\right)}{v_{coast}}\right)}^{\sigma }\right.\right.\\ \left.\left.-{\left(\frac{s_{0}+v_{i,error}\left(t\right)T+\frac{v_{i,error}\left(t\right)\Delta v_{i,error}\left(t\right)}{2{\sqrt{a_{\max}}}^{2}}}{\Delta x_{i,error}\left(t\right)}\right)}^{2}\right),a_{\min}\right\} \end{array}\right.$$

The first equation of Equation (6) limits the speed to be positive or zero, which ensures the authenticity of the extension IDM model. The second equation of Equation (6) gives the constraint of the minimal acceleration by $a_{min}$, which avoids the unrealistic braking maneuvers to be executed by the vehicle. $s_{0}$ is the allowed minimum inter-vehicle distance, and *T* is the desired time headway which indicates the time taken for the following vehicle to cover the distance to its leader, where a shorter time headway signifies closer spacing between vehicles. σ is the free acceleration exponent which determines the changing rate of the ego vehicle’s acceleration based on speed (e.g., σ=1 indicates a linear decrease, and σ→∞ corresponds to a constant acceleration). $v_{coast}$ is a preset constant which represents the desired speed of the vehicle when it is outside the V2I range and driving independently.

In the case of perceptual errors caused by vehicle controllers and sensors, the information sensed by the vehicle may randomly fluctuate around the real data. Thus, the mean-reverting process called the Ornstein–Uhlenbeck (OU) process $\varepsilon_{t}$ is used to describe the deviation of perception data from real data. The velocities $\left(v_{i}\left(t\right),v_{i-1}\left(t\right)\right)$ and inter-vehicle distance $\Delta x_{i}\left(t\right)$ are represented by the error value $\left(\varepsilon_{t_{i,1}}v_{i}\left(t\right),\varepsilon_{t_{i,2}}v_{i-1}\left(t\right)\right)$ and $\varepsilon_{t_{i,3}}\Delta x_{i}\left(t\right)$, where the OU processes $\varepsilon_{t_{i,1}}$, $\varepsilon_{t_{i,2}}$ and $\varepsilon_{t_{i,3}}$ are independent from each other to characterize the error of speed and distance. Specifically, when the value of $\varepsilon_{t_{i,1}}$, $\varepsilon_{t_{i,2}}$ and $\varepsilon_{t_{i,3}}$ are fixed to 1, the perception is completely accurate. On the other hand, as the value of OU process deviates more from 1, the CAV suffers more serious perception error due to the large difference between perception value and real value.

An OU process $\varepsilon_{t}$ in continuous time can be described as

(7) $$\begin{array}{c} \frac{d\varepsilon_{t}}{dt}=\varphi \cdot \left(\mu -\varepsilon_{t}\right)+\delta \cdot \frac{dW_{t}}{dt} \end{array}$$

where μ is a constant which determines the desired long-term mean value of the process. φ controls the degree of mean regression, where the larger φ is, the closer the real mean value of OU process is to μ. $W_{t}$ denotes a one-dimension standard Brownian motion, and δ is the weight parameter that controls the fluctuation of $\varepsilon_{t}$.

Based on [39], an OU process can be obtained by an iterative equation in discrete time $t_{k}\left(k=0,1,2,\ldots \right)$ for simulation, which can be given by

(8) $$\begin{array}{c} \varepsilon_{t_{k+1}}=h\cdot \varepsilon_{t_{k}}+\mu \cdot \left(1-h\right)+\delta \cdot \sqrt{\frac{1-h^{2}}{2\varphi }}\cdot Z_{k+1} \end{array}$$

where $h=e^{-\varphi \Delta t}$ and Δt is the minimum time step for data update. Parameters φ, μ, and δ have the same effect as illustrated in Equation (7). $Z_{k+1}$ is a sequence of standard normal random variables to introduce stochastic into the OU process. In this study, the φ, μ, and initial value $\varepsilon_{t_{0}}$ are set to 1. Parameter δ is defined as the perception error size because it controls the fluctuation of the OU process and the deviation of the perception value from the real value.

## 3. Vehicle Perception Data Processing

Based on the vehicle perception data, the vehicle controller can make the action decisions accordingly. In this section, the time that the vehicle arrives at the intersection is estimated, and then the vehicle role transition scheme is presented. Notably, a vehicle perception data fusion scheme is proposed to obtain the accurate velocity and inter-vehicle distance which reduce the accident rate due to the perceptual error and enhance the efficiency of passing through the intersection.

### 3.1. Calculation of Time-to-Arrival

#### 3.1.1. Leader Vehicle’s Time-to-Arrival

Let $t_{c}$, $t_{e}$ and $t_{l}$ denote cruising time-to-arrival, earliest time-to-arrival, and latest time-to-arrival, respectively. Assume that $v_{max}$ is the road speed limit, and $t_{i,s}$ is the time that vehicle *i* receives the information from the intersection. Based on the vehicle dynamics, $t_{c}$, $t_{e}$, and $t_{l}$ can be calculated by [37]

(9) $$\begin{array}{c} t_{c}=t_{i,s}+\frac{d}{v_{i}\left(t_{i,s}\right)} \end{array}$$

(10) $$\begin{array}{c} t_{e}=t_{i,s}+\frac{d-v_{i}\left(t_{i,s}\right)\cdot \frac{\pi }{2n}}{v_{\max}}+\frac{\pi }{2n} \end{array}$$

(11) $$\begin{array}{c} t_{l}=t_{i,s}+\frac{d-v_{i}\left(t_{i,s}\right)\cdot \frac{\pi }{2m}}{v_{coast}}+\frac{\pi }{2m} \end{array}$$

where $n=\min\left\{\frac{2\cdot a_{\max}}{v_{\max}-v_{i}\left(t_{i,s}\right)},\sqrt{\frac{2\cdot jerk_{\max}}{v_{\max}-v_{i}\left(t_{i,s}\right)}}\right\}$ and $m=\min\left\{\frac{2\cdot a_{\max}}{v_{i}\left(t_{i,s}\right)-v_{coast}},\sqrt{\frac{2\cdot jerk_{\max}}{v_{i}\left(t_{i,s}\right)-v_{coast}}}\right\}$, which are constraints due to the vehicle engine.

Given the current available green window $T_{g\_available}$, time-to-arrival $t_{arr}$ is divided into four categories. If $t_{c}\in T_{g\_available}$, then the scenario should be cruise scenario and the time-to-arrival $t_{arr}=t_{c}$; if $\left[t_{e},t_{c}\right]\cap T_{g\_available}\neq \phi$, it means that the vehicle arrives at the intersection within the available green window by accelerating. Then, $t_{arr}=\min\left[t_{e},t_{c}\right]\cap T_{g\_available}$. On the other hand, if $\left[t_{c},t_{l}\right]\cap T_{g\_available}\neq \phi$, it means that the vehicle can reach the intersection at the start time of the next green window by decelerating to avoid the current red window, and $t_{arr}=\min\left[t_{c},t_{l}\right]\cap T_{g\_available}$. Finally, if the vehicle could not pass the intersection in a green window by accelerating or decelerating, the vehicle will stop at the intersection and $t_{arr}=t_{next\_s}$, where $t_{next\_s}$ is the time to the start of the next green window.

#### 3.1.2. Follower Vehicle’s Time-to-Arrival

Followed by [20], since the vehicles in the platoon have the same time headway, we can estimate the arrival time of followers based on its order in the platoon. Firstly, a $t_{arr\_temp}$ will be calculated by $t_{arr\_temp}=\left(p-1\right)\cdot T+t_{arr\_l}$, where *p*(p=2,3,…) denotes the follower’s sequence in the platoon, and $t_{arr\_l}$ is the leader’s arrival time to the intersection obtained by V2V.

Given the current green window $T_{g\_current}$ and the next green window $T_{g\_next}$, there are four cases as follows. If $t_{arr\_temp}\in T_{g\_current}$, it means that the follower can pass the intersection in the current green phase and $t_{arr}=t_{arr\_temp}$. If $t_{arr\_pre}\in T_{g\_current}$ and $t_{arr\_temp}\notin T_{g\_current}$, the follower will be stuck due to red light. To prevent congestion at the intersection, the vehicles are expected to reach the intersection in the next green window, which can be achieved via controlling the velocity of the vehicle. Consequently, the vehicle becomes the leader of platoon and $t_{arr}=t_{next\_s}$. On the other hand, if $t_{arr\_pre}\in T_{g\_next}$ and $t_{arr\_temp}\in T_{g\_next}$, the both vehicles are still in a platoon, and $t_{arr}=t_{arr\_pre}+T$.

### 3.2. Vehicle Role Transition

#### 3.2.1. CAV Role Transition

Since the CAV vehicle can transform from a leader to a follower in a platoon, or vice versa, then we define some criterion to determine the CAV’s role. When the CAV enters the road, it continuously checks whether there is a preceding vehicle on the same lane. If no, then it becomes a leader of a new platoon and keeps a desired coasting speed $v_{coast}$ before entering the V2I range. If yes, then it has different behavior according to the type of the preceding vehicle.

For example, if the preceding vehicle is a conventional vehicle, the longitudinal behaviors are captured by the extension IDM model, where the CAV will always follow the preceding vehicle. On the other hand, when the leader is a CAV and the ego vehicle is out of the V2I range, the ego vehicle should follow the leader. Then, when they run into the V2I range, the ego vehicle will compare the time-to-arrival $t_{arr}$ and $t_{arr\_pre}$ to the intersection with the time of green window, where $t_{arr}$ and $t_{arr\_pre}$ correspond to the arrival time of the ego vehicle and the preceding vehicle, respectively. If the time difference between these two consecutive CAV vehicles is larger than the total length of a red phase, the ego vehicle will be considered as the ’string breaker’ and becomes a leader. Otherwise, the ego vehicle should always follow the preceding CAV vehicle.

#### 3.2.2. Conventional Vehicle Role Transition

For a conventional vehicle, if the distance to the intersection is longer than the visual range *H*, the vehicle will follow the pre-vehicle while there is one, or keep a desired coasting speed $v_{coast}$ while not. On the contrary, if the distance to the intersection is less than the visual range *H*, the driver can recognize the signal light and estimate the time-to-arrival $t_{arr}$. Then, the vehicle will perform the role transition, which is similar to the CAV.

### 3.3. Vehicle State Estimation Scheme

**Figure 2:**
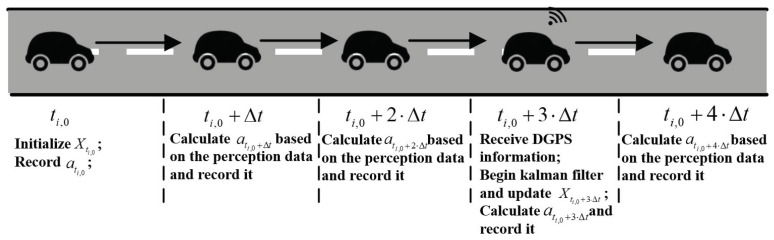
Vehicle state update process.

As described in Figure 2, vehicle *i* enters the road at $t_{i,0}$, and it first initializes state vector $X_{t_{i,0}}$ as ${\left[\begin{array}{cc} x_{i,kal}\left(t_{i,0}\right) & v_{i,kal}\left(t_{i,0}\right) \end{array}\right]}^{T}$, where $x_{i,kal}\left(t_{i,0}\right)$ and $v_{i,kal}\left(t_{i,0}\right)$ denote the position and the velocity which are set to 0 and $v_{i}\left(t_{i,0}\right)$, respectively. Then, the initial acceleration is denoted by $a_{t_{i,0}}$. To the best of our knowledge, the CAV vehicle can obtain the position and distance information via on-board sensor and DGPS, for which data update frequency is 20 HZ and 5 HZ, respectively [35]. For on-board sensors, the vehicle can obtain the data quickly and update the longitudinal control in real time, but the accuracy of sensor depends on the external conditions (e.g., weather), and errors may exist. On the other hand, the vehicle can receive the more accurate data via DGPS, but the data update frequency is slow and perception data may be outdated. To solve this challenge, a data fusion scheme for which the DGPS data interpolate on-board sensor data by the Kalman filter is presented, which enhances the accuracy of the received vehicle state and updates the vehicle information quickly.

The data fusion scheme can be implemented in two parts: prediction stage based on historical data and update stage by the currently acquired data.

#### 3.3.1. Prediction Stage

The vehicle can obtain the vehicle’s speed and acceleration value via on-board sensors. Based on the vehicle kinematic model, the vehicle state vector can be expressed by

(12) $$\begin{array}{c} X^{-}=F\cdot X_{0}+U \end{array}$$

where $X^{-}$ and $X_{0}$ represent the predicted state vector and initial state vector, respectively. *F* is the state transition matrix derived from the kinematic formula, which is $\left[\begin{array}{cc} 1 & \Delta t\\ 0 & 1 \end{array}\right]$. *U* indicates the external input value, which is related with the vehicle acceleration.

Note that Equation (12) may iterate multiple times according to the number of recorded acceleration values during the prediction period. For instance, to predict the state at $t_{i,0}+3\cdot \Delta t$ in the scenario showed in Figure 2, a vehicle can first calculate $X_{t_{i,0}+\Delta t}^{-}$ using $a_{t_{i,0}}$ and $X_{t_{i,0}}$, which can be given by

(13) $$\begin{array}{cccc} X_{t_{i,0}+\Delta t}^{-} & = & \; & F\cdot X_{t_{i,0}}+U_{t_{i,0}}\\ \; & = & \; & \;\left[\;\begin{array}{cc} 1 & \Delta t\\ 0 & 1 \end{array}\;\right]\;\cdot \;\left[\;\begin{array}{c} x_{i,kal}\left(t_{i,0}\right)\\ v_{i,kal}\left(t_{i,0}\right) \end{array}\;\right]\;+\;\left[\;\begin{array}{c} \frac{1}{2}\Delta t^{2}\\ \Delta t \end{array}\;\right]\;\cdot \;a_{t_{i,0}}\\ \; & = & \; & \;\left[\begin{array}{c} x_{i,kal}\left(t_{i,0}\right)\;+\;v_{i,kal}\left(t_{i,0}\right)\;\cdot \;\Delta t\;+\;\frac{1}{2}\cdot a_{t_{i,0}}\cdot \Delta t^{2}\\ v_{i,kal}\left(t_{i,0}\right)+a_{t_{i,0}}\;\cdot \;\Delta t \end{array}\right] \end{array}$$

Then, $X_{t_{i,0}+2\cdot \Delta t}^{-}$ can be estimated by Equation (12) with the initial state matrix $X_{t_{i,0}+\Delta t}^{-}$. In this manner, $X_{t_{i,0}+3\cdot \Delta }^{-}t$ can also be obtained by further iterations based on the previous value $X_{t_{i,0}+2\cdot \Delta t}^{-}$. The $a_{t_{i,0}}$, $a_{t_{i,0}+\Delta t}$, and $a_{t_{i,0}+2\Delta t}$ can be recorded by the vehicle controller, and cleared after a prediction process.

However, the prediction model based on on-board sensor data may not be completely accurate due to the existing perceptual error. To describe the prediction error, a covariance matrix *P* is used to measure the offset of the predicted value from the true value. An incredible matrix $Q_{0}$ is introduced to describe the credibility of the prediction model, which is set to $\left[\begin{array}{cc} 0.1 & 0\\ 0 & 0.1 \end{array}\right]$. Note that the smaller the value of $Q_{0}$, the more reliable the model. Since the perceptual error is random variance, $P_{0}$ cannot be known in advance. Based on [40], the predicted $P^{-}$ can be obtained via the iterative process to continuously approximate the true offset value, which can be given by

(14) $$\begin{array}{c} P^{-}=FP_{0}F^{\prime }+Q_{0} \end{array}$$

where $F^{\prime }$ is the transposition matrix of *F*.

#### 3.3.2. Update Stage

Based on the on-board sensor’s data, we can predict the vehicle position information. However, the predicted data may have large errors. In this subsection, the DGPS data are utilized to fuse with on-board sensors to measure the vehicle’s real-time position. Based on the Kalman filter, the updated vehicle state can be described by

(15) $$\begin{array}{c} X=X^{-}+K\left(Z-HX^{-}\right) \end{array}$$

where *K* is the Kalman gain, and *H* is the observation matrix that is used to extract the value of the predicted vehicle position from the state matrix $X^{-}$ to compare with the actual measured position *Z*. Then, the value of *H* is set to $\left[\begin{array}{cc} 1 & 0 \end{array}\right]$. Note that the difference $\left(Z-HX^{-}\right)$ is the measurement residual, which reflects the discrepancy between the predicted vehicle position $HX^{-}$ and the actual measured position *Z*.

Similar to the $X^{-}$, the predicted covariance matrix $P^{-}$ is also updated for the next filtering process by

(16) $$\begin{array}{c} P=\left(I-KH\right)P^{-} \end{array}$$

where *I* is an identity matrix.

Based on [40], the Kalman gain *K* is calculated by the error of prediction and measurement, which can be expressed as

(17) $$\begin{array}{c} K=P^{-}H^{\prime }/\left(HP^{-}H^{\prime }+R\right) \end{array}$$

where $H^{\prime }$ indicates the transposition matrix of *H*, and *R* is the DGPS measurement error.

For instance, when *R* approaches zero, $\lim_{R\to 0}K=H^{-1}$ by Equation (17), which means that the predicted value is heavily dependent on the residual and the measurement is more reliable. Based on Equation (15), we can obtain $X\overset{K\to H^{-1}}{=}H^{-1}Z$. Due to the accurate measurement, the difference between *X* and vehicle real state is very small, and the value of *P* tends to zero. However, if the estimated error $P^{-}$ approaches zero, then $\lim_{P^{-}\to 0}K=0$, and $P\overset{K\to 0}{=}P^{-}$. Because the measurement value is trustless, the value of *X* is largely determined by the $X^{-}$.

## 4. Performance Evaluation Model

In this section, we define the evaluation model to measure the performance of transportation in mobility, safety, and sustainability based on simulation data.

### 4.1. The Traffic Flow per Time

For a traffic system, the traffic flow per time defines the number of vehicles that approached the intersection in a fixed period of time, which indicates the urban transportation efficiency. Based on [26], the traffic flow per time *Q* can be given by

(18) $$\begin{array}{c} Q\;=\;\frac{card\{i\in M,\exists t⩽T_{sim}:x_{i}\left(t\right)=L\}}{T_{\mathrm{sim}}} \end{array}$$

where card denotes cardinality, *M* is the collection of vehicles, *L* is the length of the road, and $T_{sim}$ is the simulation time, which allow all vehicles to pass the traffic intersection within the simulation time. The numerator is the total number of vehicles that successfully passed the intersection within the simulation time. The number of vehicles effectively passing the intersection is closely related to the time headway *T* and perceptual error size δ. A small *T* results in a more compact platoon structure, which requires a high level of timely vehicle speed changes and increases the risk of collisions due to perceptual errors, thus reducing the number of successful vehicles.

### 4.2. The Number of Crashed Vehicles per Time

The traffic safety is reflected by the proportion of vehicles damaged by traffic accidents. For i∈M, let $P_{i}\left(t\right)=\left[x_{i}\left(t\right)-\frac{l}{2},x_{i}\left(t\right)+\frac{l}{2}\right]$ denote the area of the road which is occupied by the vehicle *i* at time t>0. The accident occurs under the condition that $B=\{i,j,t|i,j\in M,i\neq j,t>0,P_{i}\left(t\right)\cap P_{j}\left(t\right)\neq \emptyset \}\neq \emptyset$. Then, the number of crashed vehicles per time *A* can be given by

(19) $$\begin{array}{c} A=\frac{card\{i\;\in M\;,\exists t\;⩽\;T_{sim},j\;\in \;M\;,\;i\;\neq \;j:P_{i}\left(t\right)\cap P_{j}\left(t\right)\neq \emptyset \}}{T_{sim}} \end{array}$$

Based on the extended IDM model, the vehicle driving under perceptual errors can be described dynamically. When the perception error is large and the distance between vehicles is small, the followers may show aggressive following behavior and lead to too many crashes.

### 4.3. Metrics of the Traffic Congestion

When a vehicle is stuck due to a red light or an accident, it can be described as a congestion scenario. Under the influence of perception errors, vehicles that could have followed the previous platoon to pass the intersection are forced to split the platoon due to inaccurate speed control, which leads to more stops at traffic intersections and increases overall congestion times. In addition, too many accidents can also lead to traffic congestion on the road. The sum of the congestion times for all vehicles is expressed by *E*. To better describe the average congestion time of a single vehicle, a congestion function is developed to measure the severity of traffic congestion, which can be given by [41]

(20) $$\begin{array}{c} C=\frac{1}{n_{veh}}\sum_{i=1}^{n_{veh}}\eta \left[1-{\left(\frac{t_{i,wait}}{t_{tolerant}}\right)}^{\omega }\right] \end{array}$$

where $n_{veh}$ is the number of the vehicles, η and ω are constants which are 0.15 and 2, respectively. $t_{tolerant}$ is the tolerable waiting time of passenger, and $t_{i,wait}$ is the waiting time of vehicle *i*, i∈M.

### 4.4. Measurement of Vehicle Acceleration Fluctuation

The fluctuation of vehicle acceleration reflects the stability of the platoon and the comfort of passengers, which can be measured by the standard deviation of vehicle accelerations

(21) $$\begin{array}{c} f=\frac{sum\left\{i\in M,\sum_{k=1}^{n_{i,acc}}\frac{{\left(a_{i,k}-\frac{\sum_{s=1}^{n_{i,acc}}a_{i,s}}{n_{i,acc}}\right)}^{2}}{n_{i,acc}}\right\}}{Q\cdot T_{sim}} \end{array}$$

where $n_{i,acc}$ represents the number of all acceleration values recorded by the vehicle i∈M. $a_{i,k}$ or $a_{i,s}$ is the acceleration value of vehicle *i* recorded at the *k*-th or *s*-th time gap. In fact, larger time headway *T* allows for looser platoon spacing, which in turn avoids frequent speed changes and improves the error tolerance, which enhances passenger comfort and traffic safety.

### 4.5. Energy Efficiency

The energy efficiency can be reflected by the ratio of the total fuel consumption of the road over the total number of vehicles passing the intersection. Note that higher conversion efficiency corresponds to lower average fuel consumption, which can be calculated by [42]

(22) $$\begin{array}{c} F=\frac{fuel_{sum}}{Q\cdot T_{sim}} \end{array}$$

where $fuel_{sum}$ is the total fuel consumption of the road after the simulation ends. In addition, it can be given by

(23) $$\begin{array}{c} fuel_{sum}=\int_{t_{s}}^{t_{e}}c\left(v\left(t\right),a\left(t\right)\right)dt \end{array}$$

where

(24) $$c\left(v\left(t\right),a\left(t\right)\right)=\left\{\begin{array}{cc} \; & \alpha ,a\left(t\right)\;⩽-\frac{R_{a}\;\left(t\right)\;+\;R_{r}\;\left(t\right)\;}{M_{v}}\\ \; & \alpha \;+\;\beta_{1}\;R_{T}\left(t\right)v\left(t\right)\;,a\left(t\right)\;\in \;\left(-\frac{R_{a}\;\left(t\right)\;+\;R_{r}\;\left(t\right)\;}{M_{v}}\;,\;0\right)\\ \; & \alpha \;+\;\beta_{1}\;R_{T}\left(t\right)v\left(t\right)\;+\;\frac{\beta_{2}M_{v}a{\left(t\right)}^{2}v\left(t\right)}{1000}\;,a\left(t\right)\;⩾\;0 \end{array}\right.$$

and $R_{T}\left(t\right)=M_{v}a\left(t\right)+R_{a}\left(t\right)+R_{r}\left(t\right)+R_{g}\left(t\right)$ is the total energy consumption. $R_{a}\left(t\right)=\frac{\rho }{2}C_{D}A_{f}v{\left(t\right)}^{2}$ and $R_{r}\left(t\right)=0.01\frac{1+v(t)}{44.73}M_{v}g$ represent the air resistance and frictional resistance, respectively. α is a fuel consumption constant, and β is the conversion factor between energy and fuel consumption. *g* is the gravitational acceleration which means the acceleration of an object in free fall within a vacuum. ρ is the air density which is the mass per unit volume of Earth’s atmosphere. $C_{D}$ represents an air resistance coefficient which is a dimensionless quantity that is used to quantify the drag or resistance of an object in a fluid environment, such as air. It is worth mentioning that speed fluctuations due to perception errors and engine idling due to stopping at intersections can add additional energy consumption and reduce energy conversion efficiency.

## 5. Simulation Results

In this section, an intersection of one-lane road is considered for studying the relationship between *T* and δ, and the proposed vehicle state estimation algorithm is evaluating via Matlab. The length of the road is set to L=500 m. The traffic flow obeys Poisson distribution with mean traffic flow of 1500 veh/h. Meanwhile, a safety constraint is set to 7.2 m which limits the interval distance between two generated vehicles to avoid artificial accidents. As an accident occurs, the collided vehicle’s velocity is immediately set to 0 and the duration that the collided vehicles are removed from the road follows an exponential distribution, denoted by $t_{removal}∼Exp\left(\gamma \right),\gamma >0$ [26]. Note that other accidents may occur on this road at the same time and the removal time for different accident points are independent from each other. The parameters used for our simulation are given in Table 1.

### 5.1. The Effect of Vehicle Time Headway and Perceptual Error Size

Since the vehicle time headway *T* affects the safety distance of the vehicle platoon and the traffic dense on the road, we study the effect of vehicle time headway and perceptual error rate on the traffic accidents. The simulation time $T_{sim}$ is set to 700 s.

**Figure 3:**
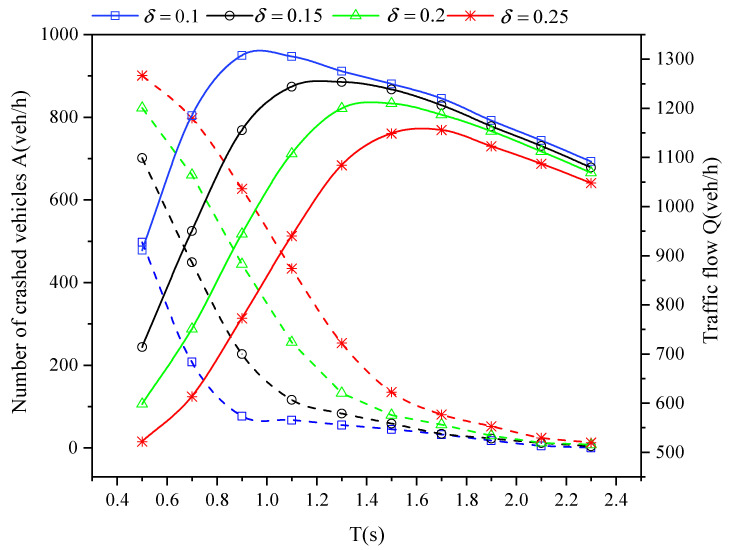
Traffic flow and crashed vehicles for varying *T* and perceptual error size δ in a completely CAV scenario with an independent on-board sensor perception scheme.

Figure 3 illustrates the comparisons between the traffic flow (solid line) and the number of crashed vehicles (dotted line) for varying *T*. It is obvious that the perceptual error will lead to more traffic accidents and less effective traffic flow when *T* is fixed, which will reduce the traffic efficiency and safety. This is because the perceptual error may lead to inaccurate speed control and the strong fluctuation of speed will be transmitted in a closed system of vehicle platoons [43], which result in more accidents. As δ is fixed, it is shown that the number of traffic flow increases firstly and then decreases. The reason for the first increase is that the looser platoon structure brings higher perceptual error tolerance, which reduce the number of accidents and increase the effective traffic flow. However, excessive *T* will lead to a larger interval between vehicles, which makes the traffic flow on the road sparser and reduces the number of vehicle passing through the intersection in limited simulation time. Therefore, in order to achieve the trade-off between vehicle safety and traffic efficiency, it is necessary to set a reasonable *T* value.

**Figure 4:**
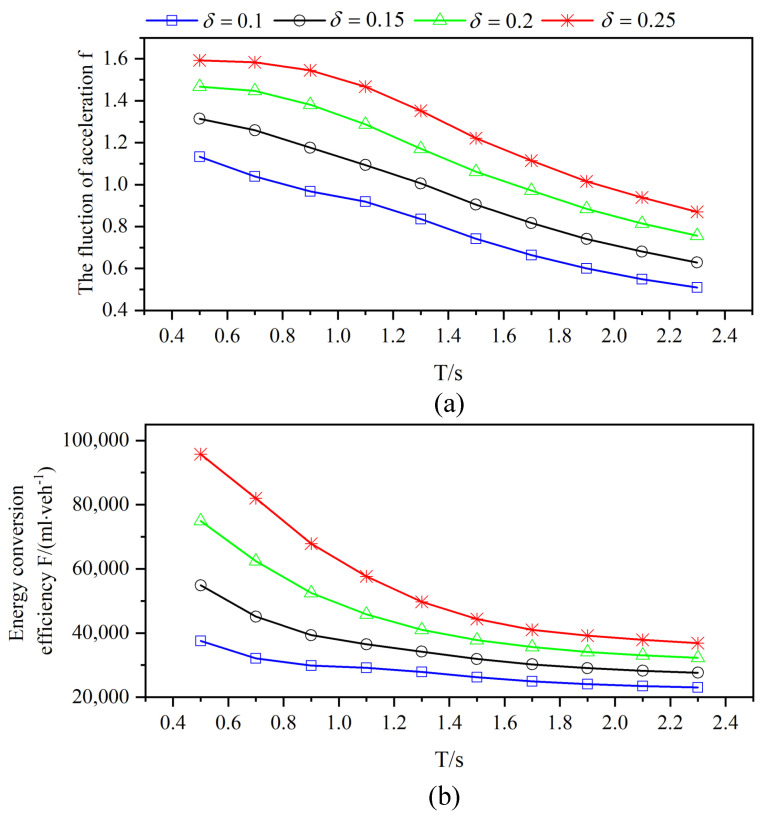
The fluctuation of vehicle acceleration (**a**) and the energy conversion efficiency (**b**) for varying *T* and perceptual error size δ in a completely CAV scenario with an independent on-board sensor perception scheme.

It is described in Figure 4 that the looser platoon structure leads to more stable acceleration fluctuation. With the smoother speed wobble, the average vehicle effective fuel consumption is gradually reduced. In particular, the optimization of energy efficiency is more significant when *T* is less than 1.4 s, and the efficiency curve tends to be stable when *T* continues to increase. The reason for the previous performance improvement can be attributed to the reduction of accidents in the first part, which leads to the rapid increase of traffic flow and brings notable improvement of energy efficiency. Then, when *T* is greater than 1.5 s, the number of effective vehicles begins to decrease from Figure 3, which makes the efficiency curve change slowly. Therefore, setting *T* to about 1.4 s can realize the balance between energy efficiency and effective vehicle flow.

**Figure 5:**
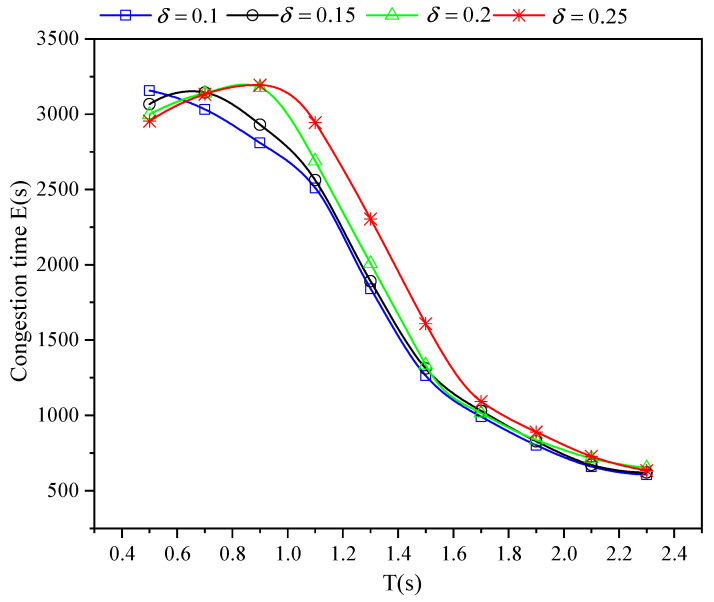
Congestion time E for varying *T* and perceptual error size δ in a completely CAV scenario with an independent on-board sensor perception scheme.

Figure 5 shows the total congestion time *E* in the simulation. Traffic may be blocked by a traffic accident or a red light ahead. With the increase of δ, the more serious perceptual error is not only caused more accidents, but also brings wobbly speed control, which interferes with CAV arriving at the intersection at an accurate speed and results in more stops at the intersection. Particularly, the larger perceptual error leads to more a serious decrease of the number of effective traffic flows when *T* < 0.7 s, which makes the total congestion time decrease with the δ increasing. In addition, the congestion time increases firstly as δ is fixed and *T* grows. It is noted that worse congestion performance is not caused by more accidents, but the sharp increasing base number of vehicles that pass through the intersection successfully. Then, when the number of accidents is less than 500 veh/h, which is 1/3 of the total traffic flow from Figure 3 at each δ. The road traffic will be smoother due to less accidents, and it will be easier for vehicles to form a long platoon to pass through signal intersections cooperatively. It shows that the performance of cooperative intersection is directly affected by the proportion of traffic accidents on the road, no matter how much perceptual error is.

### 5.2. Traffic Performance under Different Perception Schemes

#### 5.2.1. Completely CAV Scenario

In this section, we focus on the impact of different perception schemes on traffic performance in dense traffic. As shown in Figure 3 and Figure 4, when δ is fixed, the traffic performance deteriorates with the decrease of *T*, which illustrates that the effect of perceptual error becomes more serious with lower time headway and more compact distance between vehicles. Therefore, we decide to study the performance of the proposed scheme under the condition that the perceptual error is the most serious to ensure the effectiveness and robustness of the scheme. The time headway *T* is set to 0.5 s and the simulation time $T_{sim}$ is set to 700 s.

**Figure 6:**
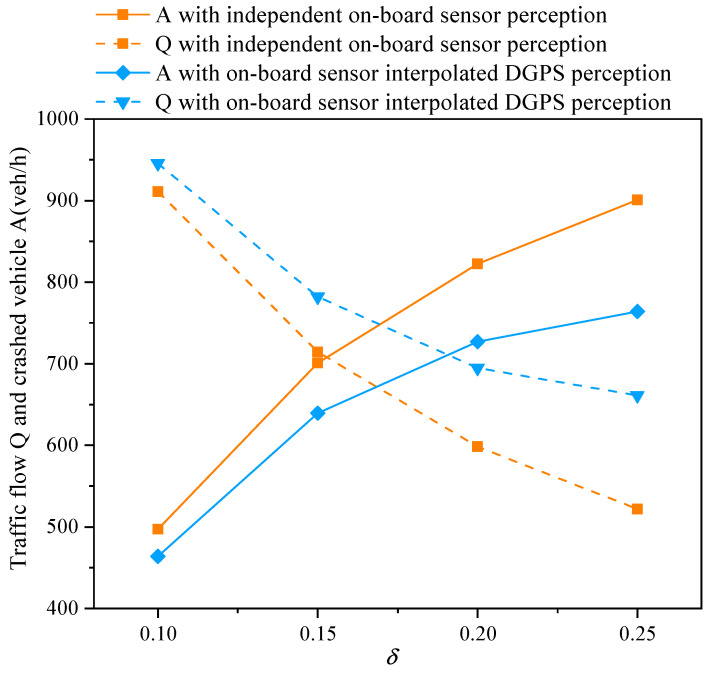
Traffic flow and crashed vehicles for varying perceptual error size δ with an independent on-board sensor perception scheme and on-board sensor interpolated DGPS perception scheme in a completely CAV scenario.

In Figure 6, comparisons between the traffic flow and the number of crashed vehicles with vehicle state estimate schemes and without schemes are presented under different perceptual errors. We can see that the number of crashed vehicles increases as the perceptual error δ increases. When the perceptual error is large, such as δ=0.25, the number of accident vehicles is nearly twice the number of vehicles that successfully pass through the intersection. Fortunately, based on the Kalman filter, the CAV can sense more accurate information by fusing sensor data and DGPS data, and execute more precise dynamics safely and quickly. The proposed data fusion scheme can greatly reduce the number of accidents, especially in the case of a large perceptual error. From Figure 6, we can obtain that the number of accidents and traffic flow are improved by 26% and 54.66%, respectively, as the δ=0.25, which can significantly improve the safety and efficiency of a transportation system.

**Figure 7:**
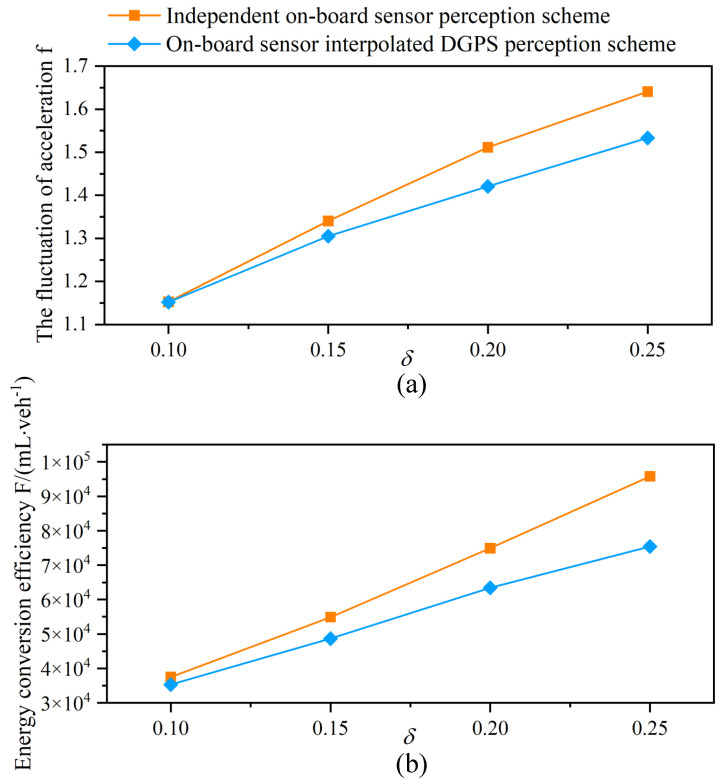
The fluctuation of vehicle acceleration (**a**) and the energy conversion efficiency (**b**) for varying perceptual error size δ with an independent on-board sensor perception scheme and an on-board sensor interpolated DGPS perception scheme in a completely CAV scenario.

Figure 7 illustrates the performance in terms of acceleration fluctuation and energy conversion efficiency of all the vehicles in simulation. Due to the error data of distance and velocity, the vehicle acceleration derived from extension IDM is inaccurate, and we can see that the speed wobble is more serious as the perceptual error δ increases. It can easily be noticed that the decrease of acceleration fluctuation is up to 5.4% under the proposed scheme, which makes the platoons more stable and improve the comfort of passengers. The reason is that accurate sensing data can be obtained by the data fusion based Kalman filter, and the cooperation between vehicles is improved. It is found that the reduction in accidents, congestion, and acceleration fluctuation by the scheme results in a significant improvement on the energy efficiency. In particular, the improvements are greatly enhanced as the perceptual error increases, which illustrate the effectiveness of the proposed scheme under high perceptual errors.

**Figure 8:**
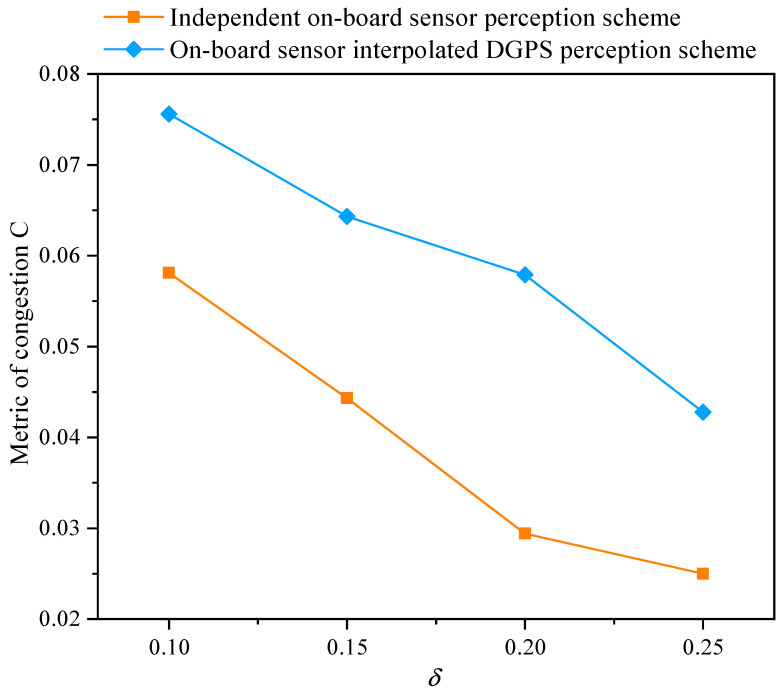
The metric of congestion for varying perceptual error size δ with an independent on-board sensor perception scheme and an on-board sensor interpolated DGPS perception scheme in a completely CAV scenario.

Figure 8 presents the effect of perceptual error on the traffic congestion. The results show that the value of *C* decreases as the perceptual error δ increases, which means that the traffic congestion is becoming serious. From Figure 8, the proposed vehicle data fusion scheme improves the congestion function *C* by an average of 61.14%, and the traffic congestion time is reduced by 9.24% based on Equation (20). Indeed, the reduction of congestion time can bring more traffic flow and truly improve the throughput at the intersection, which is essential for urban traffic during rush hours.

#### 5.2.2. Partial CAV Scenario

The effectiveness of the proposed scheme to improve the accuracy of CAV perception has been proved in the previous section. In the Partial CAV scenario, we consider heterogeneous traffic participants and study the impact of improved CAV perception accuracy on traffic under different CAV penetration rates, where the penetration rates of CAVs are set to 80%, 60%, 40%, and 20%, respectively. The time headway *T* is set to 0.5 s, and the simulation time $T_{sim}$ is set to 2000 s.

**Figure 9:**
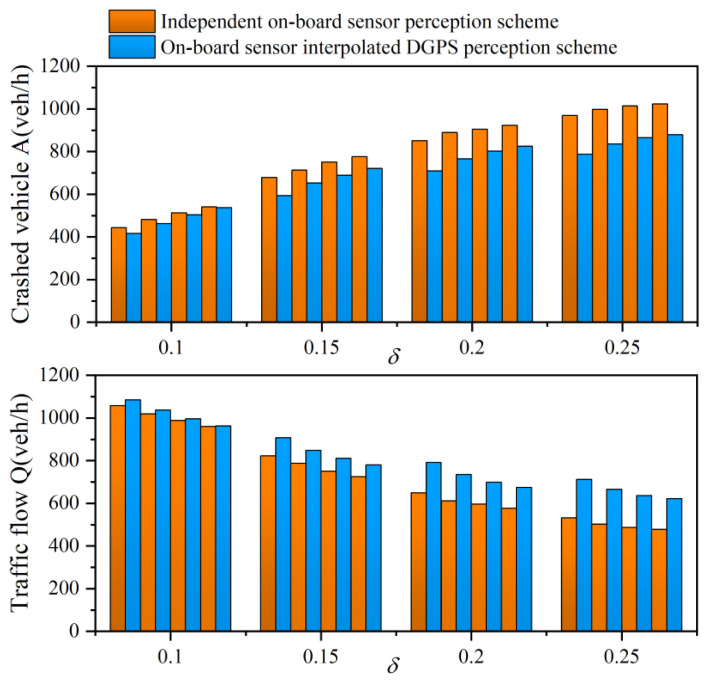
Traffic flow and Accidents for varying perceptual error size δ with an independent on-board sensor perception scheme and an on-board sensor interpolated DGPS perception scheme in a partial CAV scenario. The data correspond to 80%, 60%, 40%, and 20% CAV penetration from left to right at each δ.

Figure 9 describes the number of crashed vehicles *A* and traffic flow *Q* for varying δ and different CAV penetration rates. Actually, with increasing δ, *A* increases and *Q* decreases. When δ is fixed at 0.15 and the CAV penetration drops off, the number of accidents increases by 8.84%, 13.84%, 17.83%, and 21.13% on average compared with the completely CAV environment, respectively. The data clearly show that the CAV vehicle has positive impacts on traffic flow and accidents. The reason for the worsening accident rate can be attributed to the limitation of the conventional vehicle’s visual range, which makes the vehicle be able to only adjust the speed control near the stop bar according to the signal timing instead of the overall road. Consequently, the vehicle speed starts fluctuating, and more vehicles are gathering at the intersection, which leads to more accidents. In particular, compared with the traditional scheme, our proposed data fusion scheme shows a significant improvement on safety at different CAV penetration rates, which is up to 14.6%, 11.8%, 10.22%, and 9.62%, respectively.

**Figure 10:**
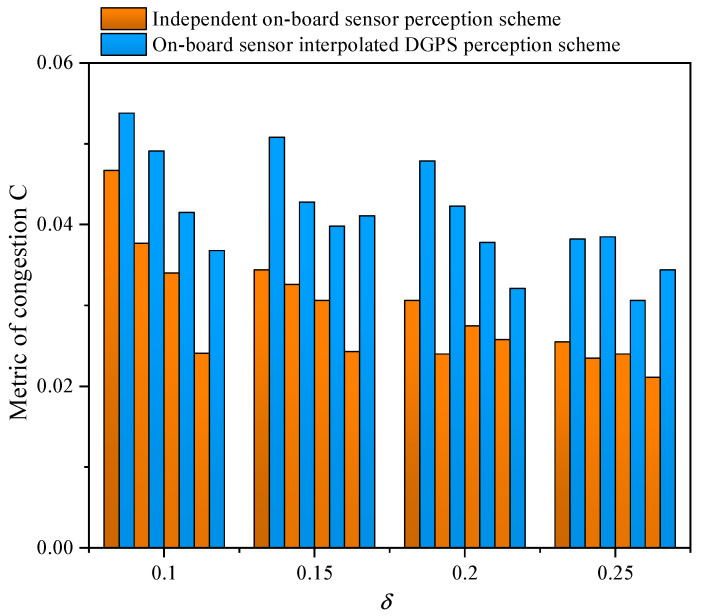
The metric of congestion function for varying perceptual error size δ with an independent on-board sensor perception scheme and an on-board sensor interpolated DGPS perception scheme in a partial CAV scenario. The data correspond to 80%, 60%, 40%, and 20% CAV penetration from left to right at each δ.

In Figure 10, the congestion becomes serious as the CAV penetration rate decreases. Compared with the independent on-board sensor perception scheme, the proposed data fusion scheme can greatly reduce congestion, especially in a partial CAV scenario. The reason can be attributed to the advantage of vehicle platoon, although conventional vehicles can not obtain SPaT information in advance, the CAV can become the leader to adjust the speed of CAV and traditional vehicles in the platoon to pass through the intersection quickly and avoid stops based on accurate sensing data. Under the CAV penetration rate, 80%, 60%, 40%, and 20%, the value of *C* can be optimized by 61.14%, 45.64%, 49.97%, and 30.62%. Based on Equation (20), the average congestion time can be decreased by 5.9%, 5.82%, 3.54%, and 5%.

**Figure 11:**
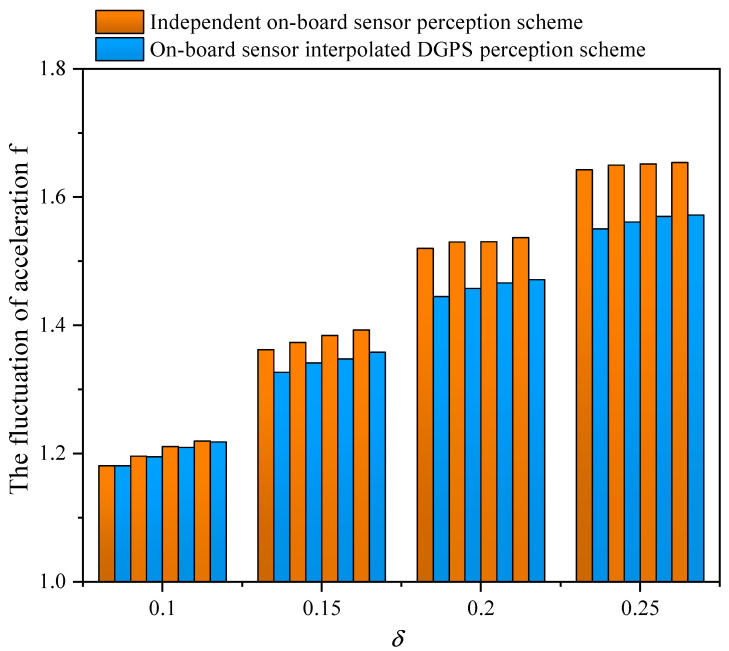
The fluctuation of vehicle acceleration for varying perceptual error size δ with an independent on-board sensor perception scheme and an on-board sensor interpolated DGPS perception scheme in a partial CAV scenario. The data correspond to 80%, 60%, 40%, and 20% CAV penetration from left to right at each δ.

Figure 11 illustrates the effect of CAV penetration rates on the vehicle acceleration fluctuation. We can see that the values of *f* are increased by 1.18%, 2%, 2.56%, and 3.03% as the CAV penetration rate decreases, which means more instability of the platoon and less comfort of the passenger. Compared with the independent sensor perception scheme, the proposed data fusion scheme decreases *f* up to 3.43% under 80% CAV penetration because accurate data perception enables CAV to better control the speed of the platoon, so as to avoid the frequent speed change of traditional vehicles at the intersection.

**Figure 12:**
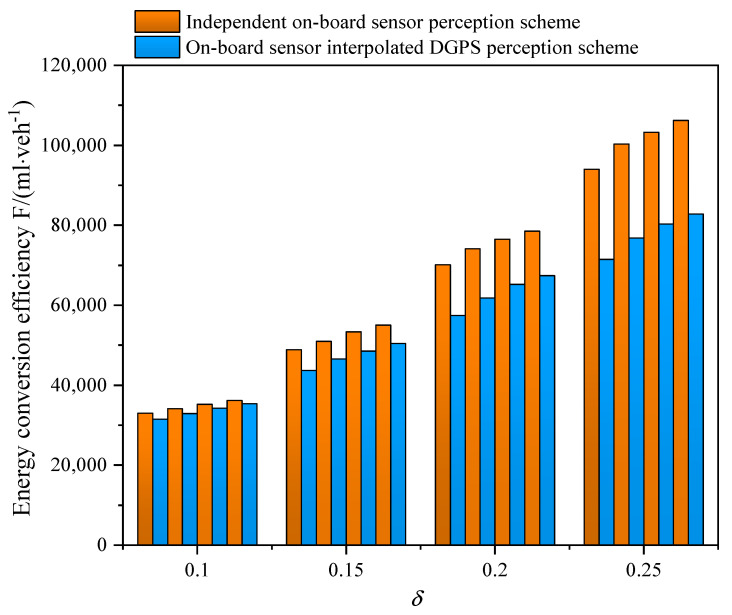
The energy conversion efficiency for varying perceptual error size δ with an independent on-board sensor perception scheme and an on-board sensor interpolated DGPS perception scheme in a partial CAV scenario. The data correspond to 80%, 60%, 40%, and 20% CAV penetration from left to right at each δ.

Figure 12 presents the performance of energy conversion efficiency under different CAV penetration rates. Apparently, the more CAV vehicles that participate in working on the road, the more fuel can be saved due to the ability of CAV to obtain signal information and adjust speed beforehand. From Figure 12, the energy conversion efficiency is improved by 17% when the CAV penetrate rate is 80%. Remarkably, the scheme optimized the energy efficiency by 14.6% on average when the CAV penetrate rate is only 20%, which indicates the effectiveness of the scheme in a low CAV penetration environment.

## 6. Conclusions

In this paper, we introduced a simulation model to study the effect of perceptual error and time headway on traffic performance in terms of safety, efficiency, and passenger comfort as well as energy conversion at cooperative intersection. Firstly, the IDM model is extended by the OU process to describe perceptual error dynamically and make the car follow mode more realistic. Secondly, the longitudinal control model is presented to capture the vehicle dynamics where vehicles can coordinate their maneuvers efficiently by switching between leader and follower and planning the optimal vehicle trajectory using a moderate acceleration to avoid red windows and reduce the number of stops. Then, we proposed a Kalman filter based data fusion scheme where the DGPS data interpolate on-board sensor data to realize accurate perception and achieve timely data updates.

Simulations have been implemented to evaluate the effect of perceptual error and time headway on the performance in traffic safety, congestion, as well as energy efficiency. The trade-off between traffic efficiency and safety are studied so that the congestion and accidents may be more serious with lower time headway and larger perceptual error size. However, excessive time headway can also reduce traffic efficiency and energy conversion due to the overly long distance between vehicles. In addition, it is found that the congestion can better be relieved when the number of crashed vehicles account for less than 1/3 of the number of total vehicles. Particularly, our proposed data fusion scheme can significantly improve the perception accuracy, and the results show that improving the perception accuracy of partial CAVs under mixed traffic flow not only optimizes the traffic flow and traffic accidents, but also ensures the passenger comfort and energy efficiency. The results facilitate an understanding of the relationship between perceptual error and traffic performance of cooperative signalized intersection in partially connected and automated traffic. In addition, the proposed model can be applied to more complex cooperative intersection scenes by introducing vehicle lateral dynamics (e.g., cooperative lane changing at intersection), which will be studied in the future.

## Figures and Tables

**Table 1:** Simulation parameters.

Notation	Definition	Value
*L*	Length of road	500 m
*l*	Length of vehicle	6 m
$n_{veh}$	Number of vehicles	200
$R_{V2V}$	Range of V2V communication	100 m
$R_{V2I}$	Range of V2I communication	300 m
$T_{switch}$	Signal switch time	60 s
α	Fuel consumption constant	0.375
β1	Resistance conversion factor	0.09
β2	Vehicle power conversion factor	0.03
Δt	Minimum time for data update	0.05 s
$a_{max}$	Maximal acceleration	2 m/s ${}^{2}$
$a_{min}$	Maximal deceleration	−3.5 m/s ${}^{2}$
$s_{0}$	Allowed minimum inter-vehicle distance	1.2 m
$jerk_{max}$	Maximal acceleration changing rate	10 m/s ${}^{3}$
$V_{max}$	Road speed limit	20 m/s
$V_{coast}$	Vehicle coasting speed	15 m/s
*g*	Gravitational acceleration	9.8 m/s ${}^{2}$
$M_{v}$	The weight of vehicle	1400 kg
ρ	Air density	1.2256 kg/m ${}^{3}$
Cd	Air resistance coefficient	0.54
$A_{f}$	Vehicle front area	2.1 m ${}^{2}$
*R*	Measurement error of DGPS	1 m
$t_{tolerant}$	Tolerable waiting time of passenger	30 s
γ	Parameters of the exponential distribution	$\frac{1}{30}$

## Data Availability

No data supporting.
